# Total wrist arthrodesis for septic wrist arthritis and loss of the bony carpus following percutaneous pinning of the fifth carpometacarpal joint: a case report

**DOI:** 10.1007/s00402-017-2660-8

**Published:** 2017-03-01

**Authors:** C. Deml, S. A. Euler, G. Schmidle, S. Erhart, M. Gabl, R. Arora

**Affiliations:** Department of Trauma Surgery, Medical University Innsbruck, Anichstrasse 35, 6020 Innsbruck, Austria

**Keywords:** Septic arthritis, Wrist, Arthrodesis, Wrist-fusion

## Abstract

We report on a patient who developed septic wrist arthritis with destruction of the entire carpus due to osteomyelitis following percutaneous pinning of a fifth metacarpal base fracture. Arthrodesis was performed using a 6 cm vascularized iliac bone graft. This case report may sharpen the surgeon’s awareness of risks in orthopedic surgeries, even though the procedure seems to be rather simple and the patient is young and seems to be healthy.

## Case report

Septic wrist arthritis (SWA) is defined as an infection of involving one or all of the radio-carpal, mid-carpal, distal radio-ulnar and carpo-metacarpal joints. In severe cases, the infection can extend into the carpal tunnel and the surrounding subcutaneous tissues [1]. Several articles are published, proposing potential pathways for the diagnosis and treatment of SWA [2]. We present a case of an end stage SWA with complete loss of the bony carpus following percutaneous pinning of the fifth carpo-metacarpal (CMC) joint, resulting in a total wrist arthrodesis. The patient was asked for submission and publication of his data and agreed.

**Fig. 1:**
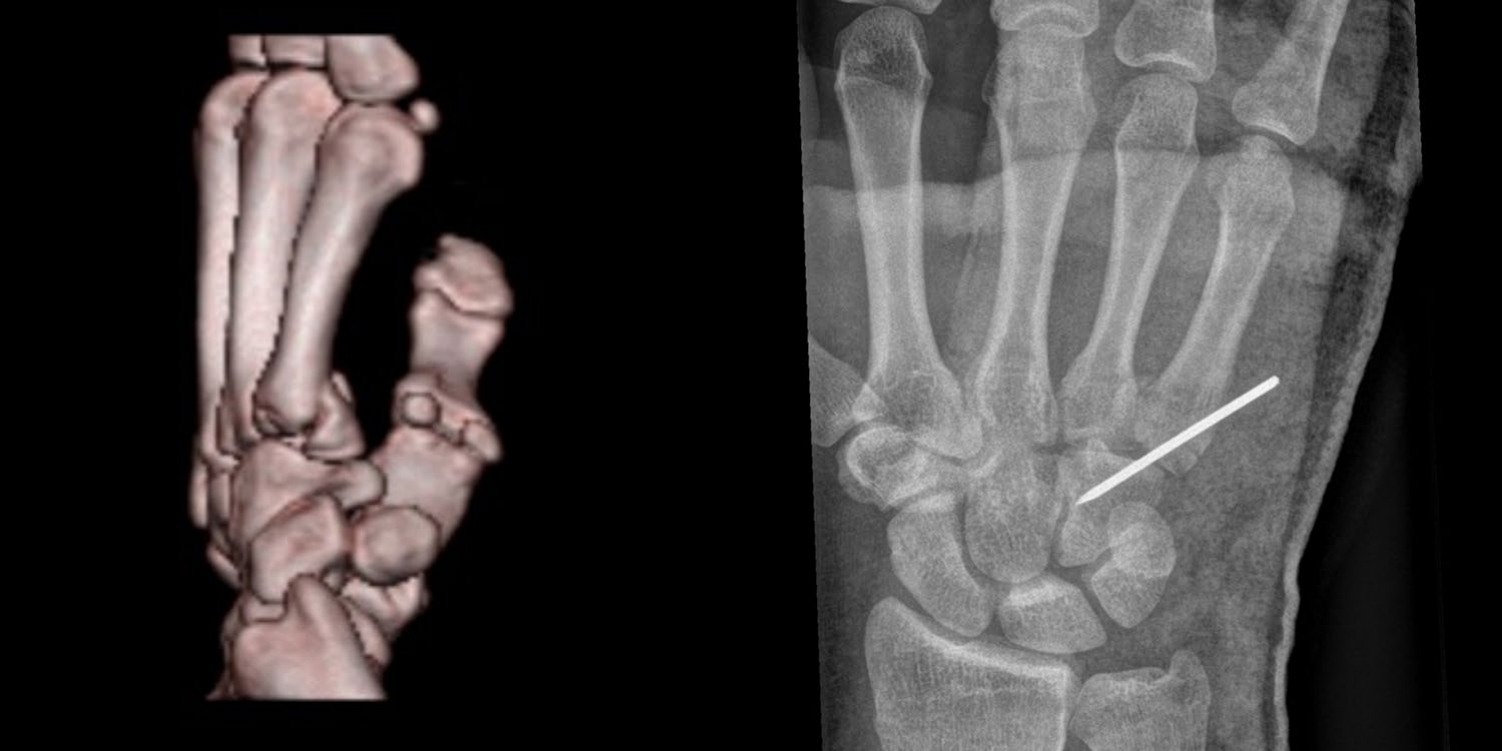
**a** Preoperative CT-scan revealing a metacarpal five fracture. **b** Postoperative p/a radiograph following pin fixation

**Fig. 2:**
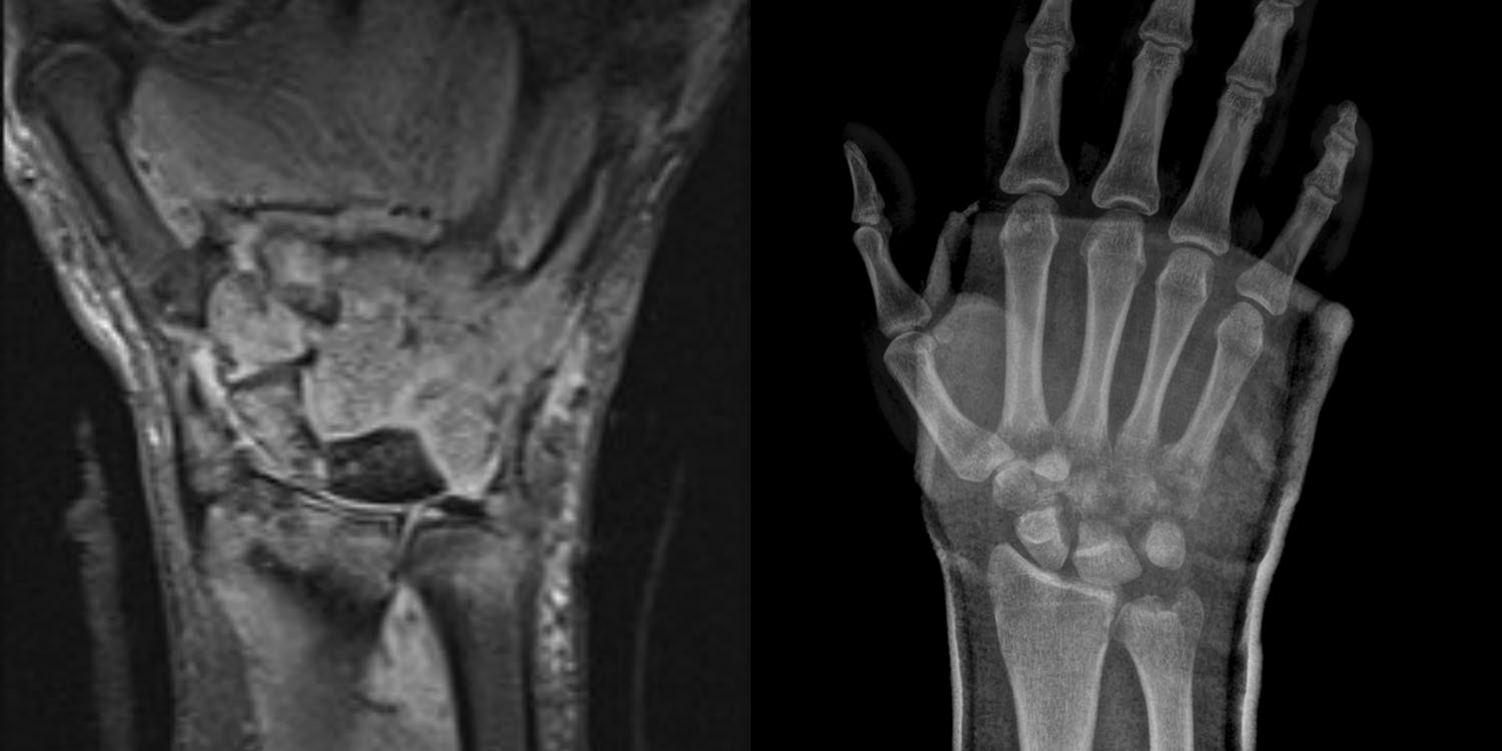
**a** MRI and **b** p/a radiograph at initial presentation in our department. Vast erosions of the wrist bones due to osteomyelitis. The MRI shows septic arthritis beginning in the bases of the metacarpal bones continuing proximally to the distal parts of radius and ulna

A 30-year-old man presented in a district hospital after a fall on ice. He injured the base of the fifth metacarpal bone and had a closed comminuted fracture. The CMC was not dislocated and the axis of the metacarpal bone was displaced palmary, 10°–15° in loss of extension. He suffers diabetes type 2. The orthopedic surgeon on call decided to surgically treat the injury using percutaneous pin fixation on the next day (Fig. 1). After the second postop X-ray at day 17, the patient developed some pain. Because of a fluctuant swelling of the hand with erythema and signs of pin infection, the pin was removed without any surgical revision. The patient was then sent home with oral antibiotics (Cefuroxim) as a “standard treatment” and scheduled for a follow-up examination. 4 weeks following pin-removal, radiographs showed a continuous infection with osteomyelitis and bony erosions of the entire carpus, including the metacarpals and the distal forearm. Finally, the patient was transferred to our University hospital. The MRI showed osteomyelitis in all carpal bones, the bases of all metacarpal bones and the distal areas of radius and ulna (Fig. 2).

Initial blood tests revealed a glucose concentration of 301 mg/dl, an HbA_1C_ of 10% and a CRP of 2.61 mg/dl. However, the white blood cell count (WBC) was found normal [8.3 (4.5–12.5) × 10^9^/l].

**Fig. 3:**
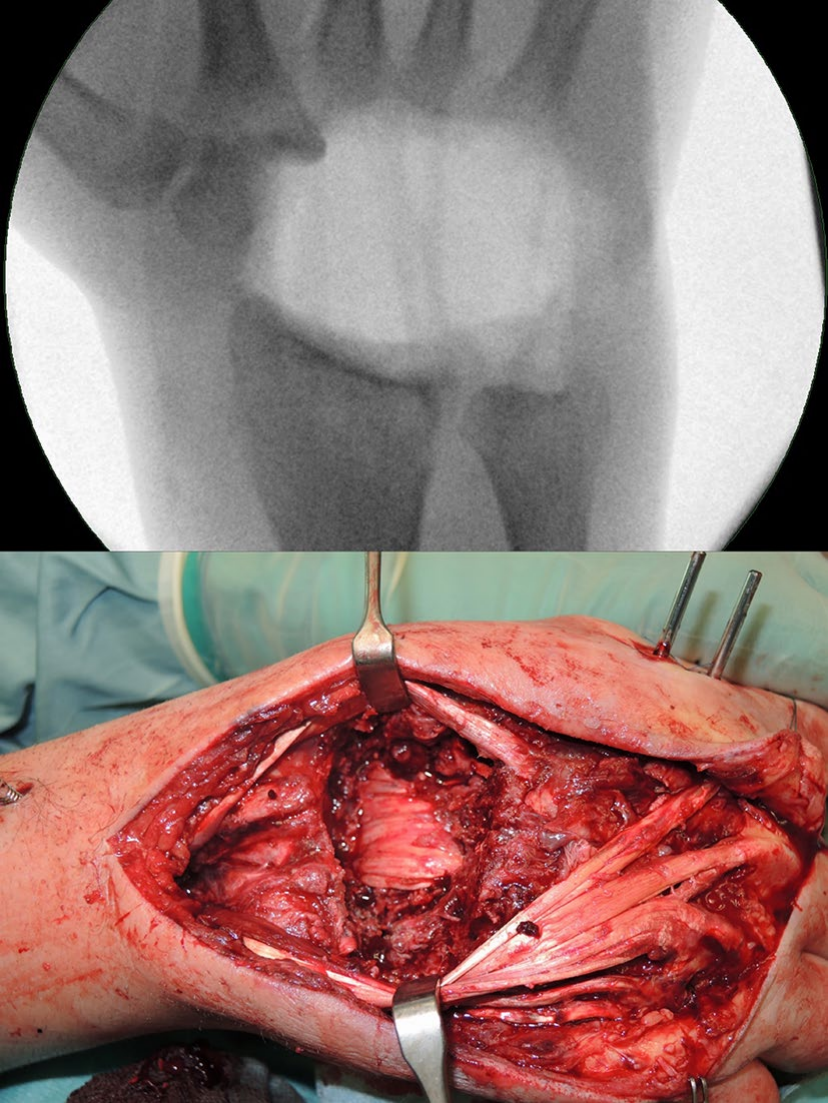
**a** Intraoperative fluoroscopy showing the complete carpal resection. **b** Surgical situs following carpectomy and meticulous soft tissue debridement

**Fig. 4:**
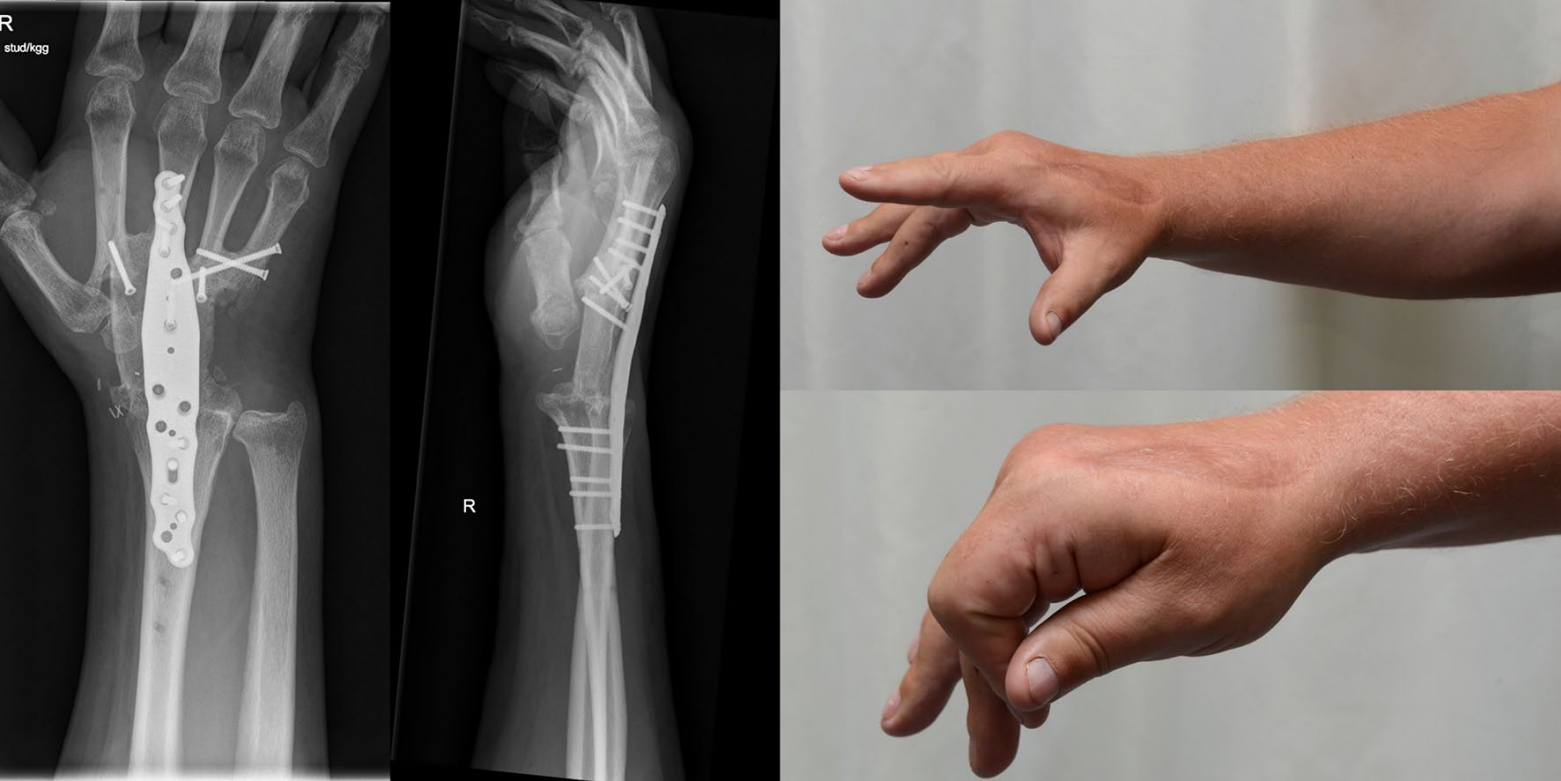
**a** 12 months follow-up radiographs showing bony union of the arthrodesis. Acceptable and stable alignment of the first digital ray without any further proximalization, resulting in a limited but satisfactory range of motion for the patient with the ability to perform a pinch grip (**b)**

Accordingly, the former pinhole and a fistula at the ulno-metacarpal area were excised in a second revision surgery. A central approach was chosen, starting between the second and third metacarpal bones, continuing proximally towards the Lister tubercle of the radius. A second fistula was found in the middle dorsum of the wrist. The subcutaneous and peritendinous tissue was fully indurated, and diffuse chambers of pus were found. The dorsal joint capsule was found to appear completely infiltrated and eroded by the infection. The wrist exhibited gross instability due to destruction of the intrinsic ligaments. Again, diffuse accumulations of pus were found in these areas. The cartilage of the carpal bones was partly destroyed, and the bony structure appeared soft and weak. All carpal bones except half of the trapezium, as well as the bases of the second to fifth metacarpal bones and the surfaces of radius and ulna had to be resected. Debridement and synovectomy of the flexor tendons were performed and the median nerve was inspected through the dorsal approach as well (Fig. 3). After meticulous irrigation and installation of an external fixator, the defect and the wounds were covered using a VAC System. Postoperatively, a diabetic specialist followed the patient daily during his stay in the hospital to maintain low blood sugar levels. The bacteriologic tests of the tissue samples collected at multiple different areas during surgery showed multi-resistant *Staphylococcus aureus* (ORSA) and *Staphylococcus epidermidis*. Antibiotic treatment using Linezolid and Fosfomycin was started immediately, according to the recommendations of the consulted University department of medical microbiology. 18 days after the initial revision surgery, followed by four revision procedures, the VAC System was finally removed, a Palacos cement spacer, saturated with Vancomyzin, was placed into the defect and the wound was closed. At this time, all collected tissue samples of the most recent revision surgery were found to be sterile and the patient was dismissed with continuous oral antibiotic therapy (Linezolid 1200 mg/d). 14 days later (32 days after initial revision), the definitive fusion of the wrist was performed, implanting a vascularized iliac bone auto graft (6 × 3 cm) connecting to the radial artery. Necrotic wound margins of 3 × 0.5 cm necessitated a visor flap from radial and split skin grafting. Bony healing was achieved after 3 months, and acceptable mobility of the thumb and index finger were accomplished after 12 months (Fig. 4).

## Discussion

We present a severe case of SWA, leading to a resection of the complete bony carpus and a total wrist arthrodesis following pin fixation of the CMC joint. Diagnosis of SWA may be difficult, even for experienced physicians. The incidence of confirmed and likely septic arthritis in Western Europe is 4–10 per 100,000 patients per year [3, 10]. Risk factors for the development of SWA include rheumatoid arthritis,  multiple drug abuse (psychotropic drugs), chronic alcohol abuse, previous intraarticular corticosteroid injections, cutaneous ulcers and insulin-dependent diabetes, as well as a low socio-economic status [5, 8, 11]. However, SWA may also occur following a surgical intervention. Our patient suffered severe insulin-dependent diabetes. There is hardly any evidence about the type and timing of the antibiotic treatment and clear guidelines are still missing [14]. The gold standard for the treatment of SWA is the initial needle aspiration. Examination of aspirated sample should include gram stain, culture, WBC and differential and microscopy for crystals. Finding gout or pseudo gout crystals does not exclude additional infection. As a second step, the surgical intervention as well as revision surgeries if needed are recommended [1, 6, 7]. Additional antibiotic treatments should be initiated according to the generated antibiogram using the collected tissue samples of the index surgical procedure. Studies showed that the most common bacteria detected were *Staphylococcus aureus* [9, 15]. The average duration of antibiotic treatment has been explained diversely in literature. The authors describe treatment periods between 5 and 180 days [7, 15]. There is hardly any evidence about the sequence, combination, or the superiority of primary closed needle aspiration and surgical aspiration with or without arthroscopy. In early stages, arthroscopic irrigation has shown likely better outcomes than the open revision with irrigation [12]. However, due to the severe grade of osteomyelitis within the carpal bones, this was not an option within our patient. Agreement exists that the key of treatment should include early removal of any infected material in combination with an individual and selective antibiotic treatment [2].

Negative WBC and negative or only slightly elevated CRP count have been described by other authors in comparable cases of septic arthritis before [9]. This may be a potential reason for the underestimation of the early stage pathology of a septic arthritis. Therefore, it seems to be important to precisely survey all anamnestic information and to closely consider possible risk factors. The initial blood glucose level in our patient was over 300 mg/dl and the HbA_1C_ test of 10% revealed a poorly regulated insulin-dependent diabetes. In combination with postoperative clinical signs of infection, including erythema and swelling, the treating doctor must be sensitized and aware of an infection. Care should be taken in cases with slightly spotted decalcification on the radiographs in combination with limited range of motion and pain, not to misdiagnose a chronic regional pain syndrome. Additional investigations like ultrasound or MRI might be helpful to further elucidate potential early stages of infection. In our case, the radiographs of the infected hand with osteomyelitis and erosions of the entire carpus were obvious signs of the ongoing infection.

The clinical outcome after SWA is poor and disappointing in the majority of the cases. In up to 73% of the cases, SWA results in a partially destroyed joint or may even result in amputation [4, 13, 15].

This case report may sharpen the surgeon’s awareness of risks in orthopedic surgeries, even though the procedure seems to be rather simple and the patient is young and healthy.
